# Significant correlation between HSPA4 and prognosis and immune regulation in hepatocellular carcinoma

**DOI:** 10.7717/peerj.12315

**Published:** 2021-10-26

**Authors:** Bing-Bing Shang, Jun Chen, Zhi-Guo Wang, Hui Liu

**Affiliations:** 1 Second Hospital of Dalian Medical University, Dalian, China; 2 Laboratory Animal Center, Dalian Medical University, Dalian, China

**Keywords:** Antigen processing and presentation, Hepatocellular carcinoma, Heat shock protein, Immune cells, Immune checkpoints, Methylation

## Abstract

**Background:**

Hepatocellular carcinoma (HCC) is an inflammation-associated tumor involved in immune tolerance and evasion in the immune microenvironment. Heat shock proteins (HSPs) are involved in the occurrence, progression, and immune regulation of tumors. Therefore, HSPs have been considered potential therapeutic targets. Here, we aimed to elucidate the value of HSP family A (Hsp70) member 4 (HSPA4) in the diagnosis and predicting prognosis of HCC, and its relationship with immune cell infiltration, immune cell biomarkers, and immune checkpoints. Gene mutation, DNA methylation, and the pathway involved in HCC were also analyzed.

**Methods:**

The gene expression omnibus (GEO) and The Cancer Genome Atlas (TCGA) databases were used to compare HSPA4 expression, and the results were confirmed by immunohistochemical staining of clinical samples. R package was used to analyze the correlation between HSPA4 and cancer stage, and to establish receiver operating characteristic (ROC) curve of diagnosis, time-dependent survival ROC curve, and a nomogram model. cBioPortal and MethSurv were used to identify genetic alterations and DNA methylation, and their effect on prognosis. The Tumor Immune Estimation Resource (TIMER) was used to analyze immune cell infiltration, immune cell biomarkers, and immune checkpoints. The STRING database was used to analyze protein–protein interaction network information. Gene Ontology (GO) analysis and the Kyoto Encyclopedia of Genes and Genomes (KEGG) analyses were performed to investigate the functions of HSPA4 and its functional partner genes.

**Results:**

Overexpression of HSPA4 was identified in 25 cancers. Overexpression of HSPA4 considerably correlated with cancer stage and alpha-fetoprotein (AFP) level in HCC. Patients with higher HSPA4 expression showed poorer prognosis. HSPA4 expression can accurately identify tumor from normal tissue (AUC = 0.957). The area under 1-, 3-, and 5-year survival ROCs were above 0.6. The HSPA4 genetic alteration rate was 1.3%. Among the 14 DNA methylation CpG sites, seven were related to the prognosis of HCC. HSPA4 was positively related to immune cell infiltration and immune checkpoints (PD-1 and CTLA-4) in HCC. The KEGG pathway enrichment analysis revealed HSPA4 enrichment in antigen processing and presentation together with HSPA8 and HSP90AA1. We verified the value of HSPA4 in the diagnosis and predicting prognosis of HCC. HSPA4 may not only participate in the occurrence and progression but also the immune regulation of HCC. Therefore, HSPA4 can be a potential diagnostic and prognostic biomarker and a therapeutic target for HCC.

## Introduction

Hepatocellular carcinoma (HCC) is one of the most aggressive neoplasms, with a 4-year survival rate of only 15% (Bray et al., 2018; Cronin et al., 2018). In the past decades, the application of comprehensive prevention, monitoring, diagnosis, and treatment methods has effectively prevented and controlled this disease (Yang et al., 2019). However, the incidence and mortality rates are still rising. Worldwide, most patients with HCC are in an advanced stage (Global Burden of Disease Cancer Collaboration et al., 2017), which is largely attributed to the poor efficiency of existing treatments in patients with advanced cancer. Therefore, it is essential to explore new methods of treatment. The immune microenvironment of HCC affects tumor progression, invasion, recurrence, and metastasis by establishing a symbiotic relationship with cancer cells (Fu et al., 2019; Jindal, Thadi & Shailubhai, 2019). Therefore, studying the role of immune-related genes and understanding the immune microenvironment would help understand the mechanism regulating this disease and guide drug usage or treatment strategies.

In the process of cancer development, the dysregulation of heat shock proteins (HSPs) is common. In tumorigenesis, HSPs are involved in the stabilization of functions of mutated or aberrantly expressed tumor-related genes. In addition, HSPs can provide danger signals to the host’s immune system and play a part in enhancing anti-tumor immunity. Therefore, HSPs have been considered potential therapeutic targets, as proposed by Lv et al. (2012), who reported that tumor-derived exosomes that contain HSPs can be used as a therapeutic vaccine. Zhou et al. (2019) reported that inhibitors of Hsp90α can be used for treating HCC with fewer toxic adverse effects. Hu, Yang & Sang (2020) and Chen et al. (2021) included HSP family A (Hsp70) member four (HSPA4) in a risk score model that consisted of several differentially expressed immune genes to predict the prognosis for HCC. However, the risk score model was discussed as a whole in these two studies, and the potential role of HSPA4, as a single factor, on the diagnosis, prognosis, DNA methylation, immune cell infiltration, and immune checkpoint of HCC has not been described.

Here, we aimed to elucidate the value of HSPA4 in the diagnosis and predicting prognosis of HCC, and its relationship with immune cell infiltration, immune cell biomarkers, and immune checkpoints. Gene mutation, DNA methylation, and the pathway involved in the occurrence and progress of HCC were also analyzed.

## Materials & methods

### Immunohistochemical staining and ethics statement

Five pathologically diagnosed patients with HCC from the Second Hospital of Dalian Medical University were randomly selected for immunohistochemical staining to validate HSPA4 expression in HCC tissues (Anti-HSPA4 antibody (EPR14166); Abcam,Cambridge, England). Both cancerous and adjacent noncancerous tissues were collected simultaneously.

The ethics committee of the Second Hospital of Dalian Medical University approved this study in strict adherence to the Declaration of Helsinki protocol (number 2021059). Written informed consent was obtained from each patient.

### Comparison of the HSPA4 expression level

Data from the TCGA database were used to analyze the expression of HSPA4 in 26 types of human cancer, in 374 HCC tissues with 50 normal liver tissues, and in 50 HCC tissues with their paired adjacent normal liver issues. In addition, gene expression profiles GSE62232 and GSE101685 were extracted from the GEO database to analyze the expression of HSPA4.

### Correlation analysis of HSPA4 and cancer stage and prognosis

Correlation analysis of HSPA4 and cancer stage, including T stage, pathologic stage, histological grade, and AFP level was performed with R using the ggplot2 package. Kaplan–Meier plots were created and log-rank test was performed using survival package. The ROC curve of diagnosis, time-dependent curve of diagnosis, and nomogram model analysis were created using R packages, including the pROC, timeROC, and survival packages. The clinical data used in this section were retrieved from the TCGA database.

### Genetic alteration in patients with HCC

The genomic profiles of HSPA4 in cBioPortal (www.cbioportal.org) were analyzed using three datasets, namely, hepatocellular carcinomas from *TCGA, Firehose Legacy*; *AMC Hepatology 2014*; and *INSERM, Nat genet 2015*. Kaplan–Meier plots were created and log-rank test was performed to identify the significance of the difference between the survival curves, and differences with *p* < 0.05 were considered statically significant.

### DNA methylation information of HSPA4

The MethSurv database (https://biit.cs.ut.ee/methsurv/) was applied to analyze the DNA methylation sites of HSPA4 in the TCGA database. Moreover, the prognostic values of CpG methylation in HSPA4 was evaluated, and the survival outcome was the overall survival (OS).

### Correlation analysis of HSPA4 with immune cell infiltration and immune checkpoints

TIMER (https://cistrome.shinyapps.io/timer/) was used to analyze the correlation of HSPA4 expression with immune cell infiltration and immune cell biomarkers in HCC. In addition, the correlation of HSPA4 expression with immune checkpoints in HCC was evaluated using TIMER and R with the ggplot2 package based on TCGA. Results with a *p* < 0.05 were considered statistically significant.

### Gene ontology (GO) analysis and kyoto encyclopedia of genes and genomes (KEGG) analyses

The STRING database was used to obtain PPI network information of HSPA4. When the protein interaction score was above 0.9, the interaction was considered statistically significant. Ten functional partner genes were obtained for the GO term enrichment and KEGG pathway analyses to investigate the functions of HSPA4.

### Statistical methods

R (v.3.6.3) was used to perform statistical analysis. Differences between groups were compared using the Wilcoxon rank-sum test or Student’s *t*-test, as appropriate. Correlations were determined using Pearson or Spearman correlation tests, as appropriate. Kaplan-Meier plots were created and log-rank tests were performed to identify the significance of the difference between the survival curves, and differences with a *p* < 0.05 were considered statistically significant.

## Results

### Higher HSPA4 expression levels in cancerous than in noncancerous tissues

**Figure 1:**
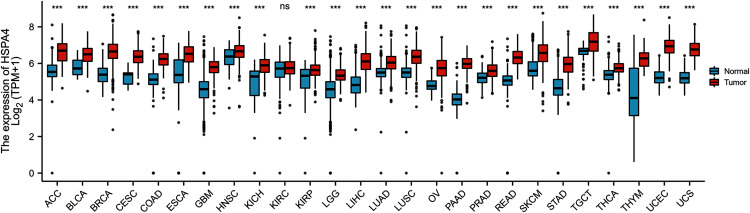
HSPA4 expression status in cancers. HSPA4 expression levels in 26 types of cancer tissues and normal tissues (TCGA cancer data compared with TCGA and GTEx normal data), ***p < 0.001; ns, no statistical difference.

To explore the possible roles of HSPA4, we first analyzed its expression in 26 types of human cancer. As shown in Fig. 1, HSPA4 was significantly unregulated in 25 cancer types as compared with the corresponding normal tissues, including bladder urothelial carcinoma (BLCA), breast invasive carcinoma (BRCA), breast invasive carcinoma (CHOL), colon adenocarcinoma (COAD), glioblastoma multiforme (GBM), kidney chromophobe (KICH), liver hepatocellular carcinoma (LICC), lung squamous cell carcinoma (LUSC), rectum adenocarcinoma (READ), thyroid carcinoma (THCA), kidney renal papillary cell carcinoma (KIRP), lung adenocarcinoma (LUAD), prostate adenocarcinoma (PRAD), and uterine corpus endometrial carcinoma (UCEC). However, no significant difference was observed in kidney renal clear cell carcinoma (KIRC).

**Figure 2:**
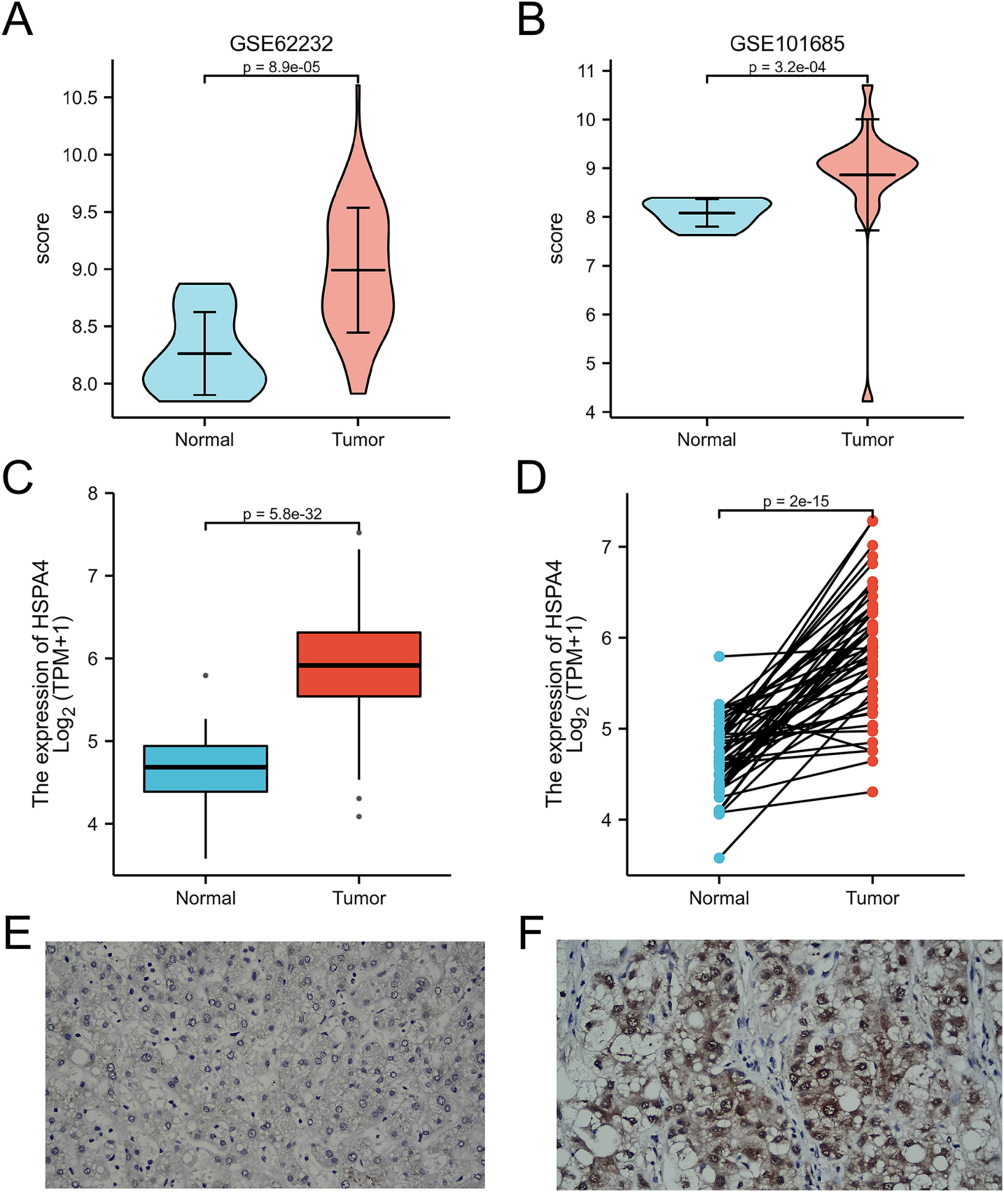
Overexpression of HSPA4 in HCC. Histogram and volcanic plot show that HSPA4 expression was higher in HCC than in the normal tissue in GSE62232 and GSE101685 (*p* < 0.001) (A, B). Based on TCGA dataset, in 374 HCC tissues and 50 normal liver tissues (C), and in 50 HCC tissues and their paired adjacent normal liver tissues (D), the expression of HSPA4 was higher in HCC (*p* < 0.001). Immunohistochemical staining of clinical HCC samples confirmed that the level of HSPA4 in tumor tissues was higher than that in its adjacent normal liver (E, F).

A higher expression of HSPA4 was observed in the GSE62232 and GSE101685 datasets (*p* < 0.001) (Figs. 2A, 2B). Overexpression of HSPA4 was observed in both paired and unmatched comparative studies based on the TCGA database (Figs. 2C, 2D). Immunohistochemical staining of clinical HCC samples also confirmed that the level of HSPA4 in tumor tissues was higher than that in adjacent normal liver tissues (Figs. 2E, 2F).

### HSPA4 expression correlated with cancer stage and prognosis

**Figure 3:**
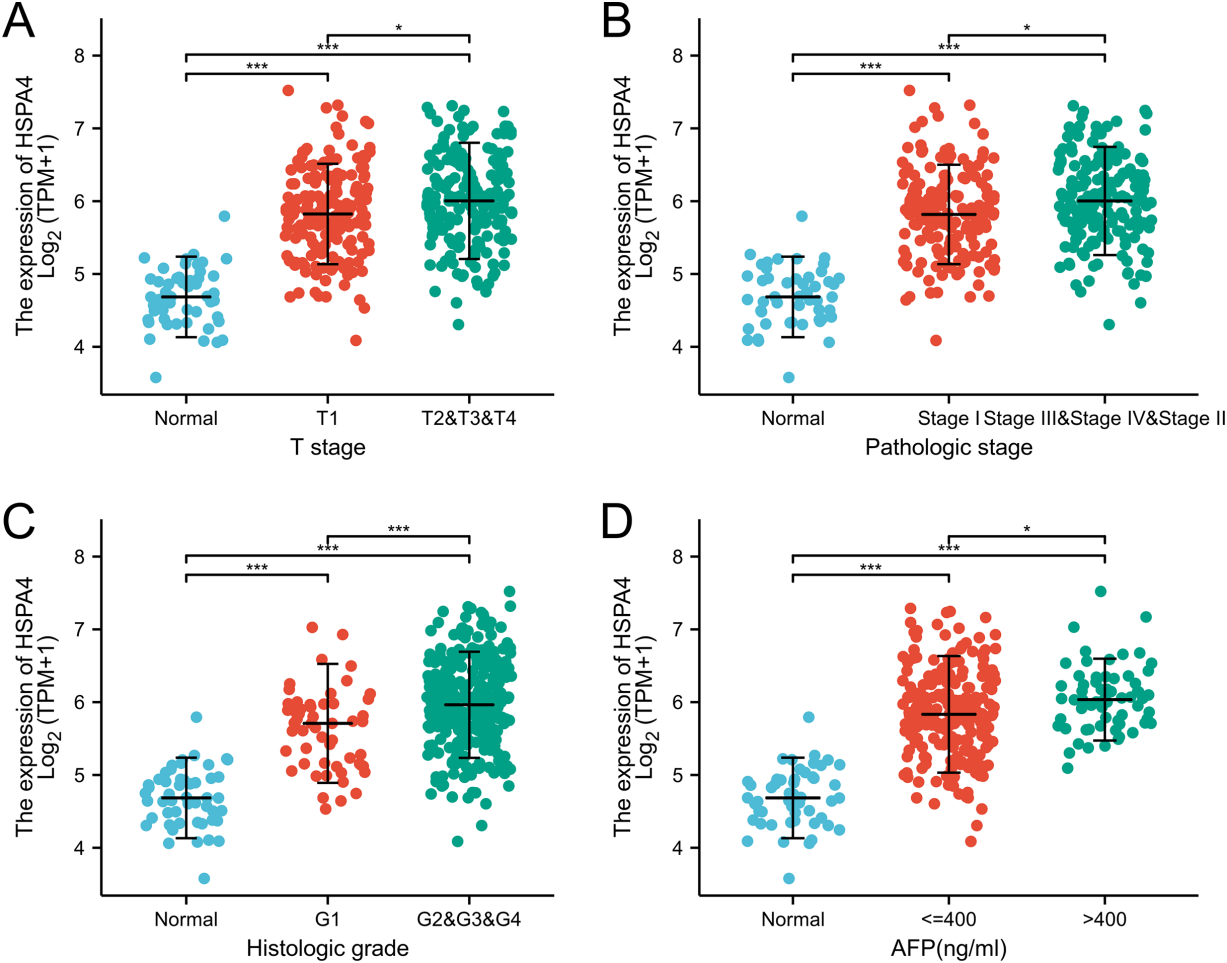
Relationship between the expression of HSPA4 and stage of HCC. The expression of HSPA4 remarkably correlated with T stage (A), pathologic stage (B), histologic grade (C), and AFP level (D). *p < 0.05, ***p < 0.001.

The expression of HSPA4 remarkably correlated with T stage (Fig. 3A), pathologic stage (Fig. 3B), and histologic grade (Fig. 3C). Patients who were in a more advanced cancer stage tended to express a higher level of HSPA4. Higher HSPA4 expression was also associated with the AFP level (Fig. 3D).

**Figure 4:**
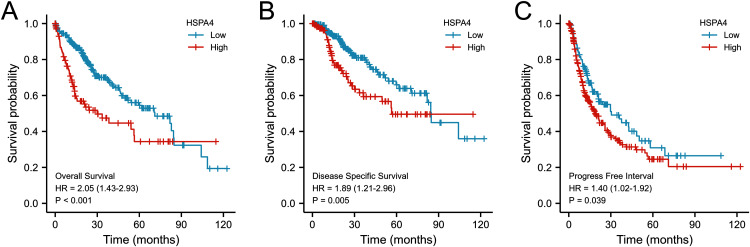
Prognostic value of mRNA level of HSPA4 in patients with HCC (Kaplan–Meier plotter). The OS (A), DSS (B), and PFI (C) survival curves comparing patients with high (red) and low (blue) HSPA4 expression in HCC were plotted at the threshold of *p* < 0.05.

According to the Kaplan–Meier survival curves, HCC cases with higher HSPA4 expression showed a lower OS (*p* < 0.001), a poorer disease specific survival (DSS) (*p* = 0.005), and a progression-free interval (PFI) (*p* = 0.039) (Fig. 4). Overexpression of HSPA4 was associated with a poorer prognosis.

### Value of HSPA4 overexpression in diagnosis and predicting prognosis

**Figure 5:**
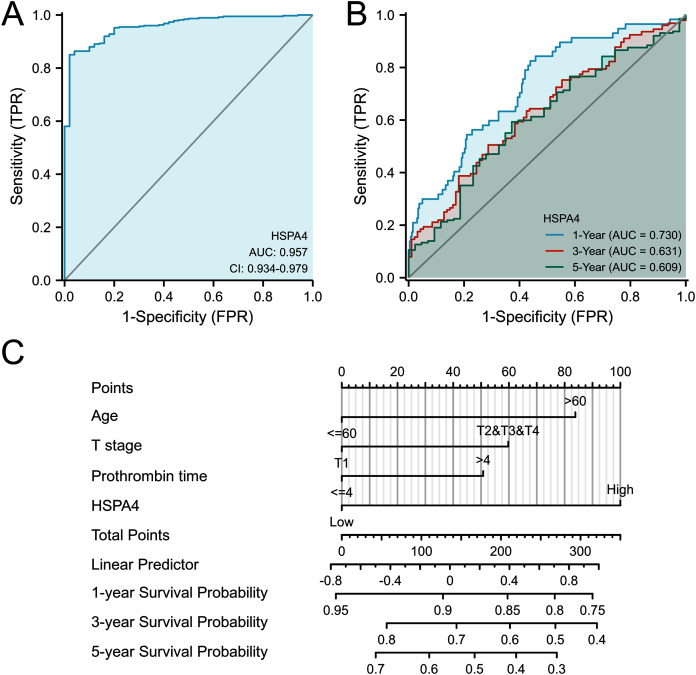
ROC analysis and nomogram model of HSPA4. (A) The ROC curve of diagnosis to distinguish tumor from normal tissue. (B) Time-dependent survival ROC curve analysis to predict 1-, 3- and 5-year survival rates. (C) Nomogram model, integrating clinicopathologic factors and HSPA4 level to predict survival probability at 1-, 3- and 5-years.

From the ROC curve of diagnosis, HSPA4 expression showed an ability to accurately identify tumor from normal tissue (AUC = 0.957) (Fig. 5A). Time-dependent survival ROC curve of HSPA4 was created to predict 1-, 3-, and 5-year survival rates. All these AUC values were above 0.6, which is considered suitable for prediction (Fig. 5B). By integrating clinicopathologic factors (including age, T stage, and prothrombin time) and the HSPA4 level, a nomogram model was built, which can be used to predict the survival probabilities at 1-, 3-, and 5-years for patients in clinical practice (Fig. 5C).

### HSPA4 genetic alteration in patients with HCC

**Figure 6:**
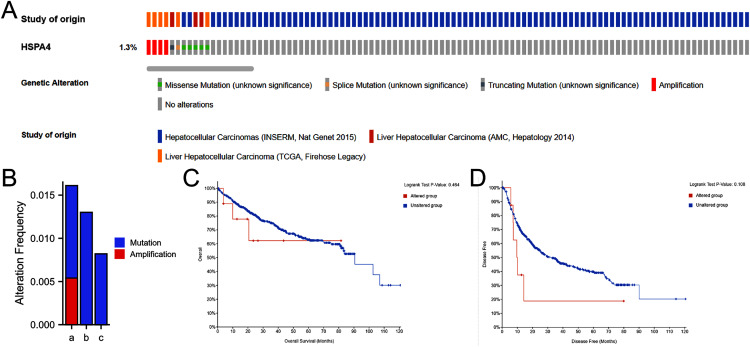
Genetic alteration in HSPA4 in HCC. (A) OncoPrint visual summary of alteration on a query of HSPA4. (B) Summary of alterations in HSPA4 in HCC from *TCGA, Firehose Legacy* (a); *AMC Hepatology 2014* (b); and *INSERM, Nat genet 2015* (c). Kaplan–Meier plots comparing OS (C) and disease-free survival (D) in patients with/without *HSPA4* gene alterations.

A total of 916 patients with HCC from three datasets were analyzed—including *TCGA*, *Firehose Legac*; *AMC Hepatology 2014*; and *INSERM*, *Nat genet 2015*. The percentage of HSPA4 genetic alterations in HCC was 1.3% (Fig. 6A), and the alteration rate ranged from 0.82 (2/243) to 1.59% (6/377) (Fig. 6B). Kaplan–Meier plots and log-rank tests indicated no significant differences in OS (*p* = 0.464) (Fig. 6C) and disease-free survival (*p* = 0.180) (Fig. 6D) between patients with alterations and those without alterations.

### HSPA4 methylation in patients with HCC

**Figure 7:**
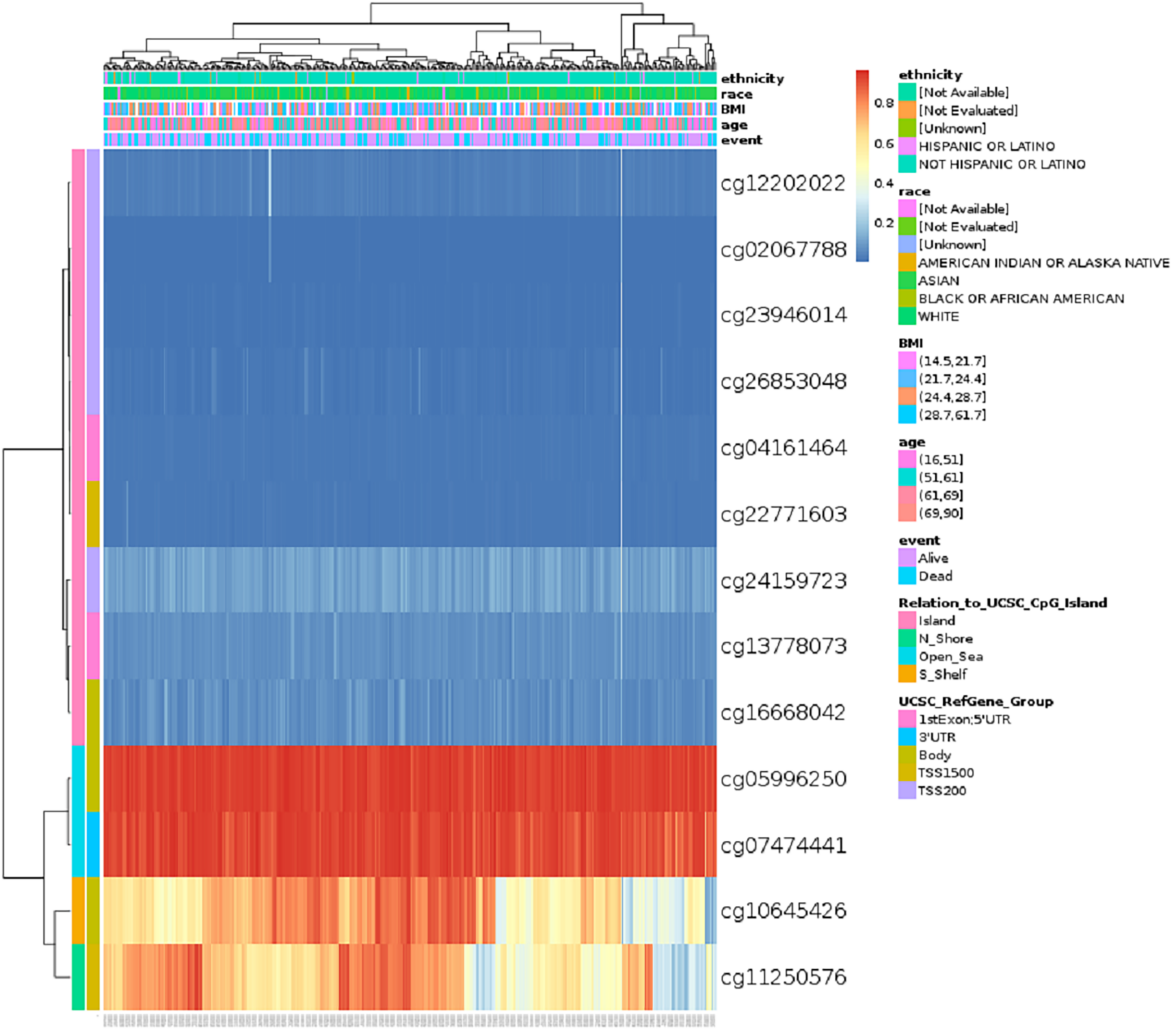
Visualization between the methylation level and HSPA4 expression.

DNA methylation levels of HSPA4 with the prognostic value of each single CpG were investigated using the MethSurv tool. The results of MethSurv suggested 14 methylation CpG sites, among which cg05996250 and cg07474441 had the highest DNA methylation (Fig. 7). The methylation level of seven CpG sites correlated with prognosis, namely, cg02067788, cg24159723, cg26853048, cg04161464, cg13778073, cg05996250, and cg07474441 (*p* < 0.05) (Table 1). Patients with high HSPA4 methylation of these CpG sites had a worse overall survival than patients with low HSPA4 methylation.

**Table 1:** Effect of hypermethylation level on prognosis in HCC.

CpG	HR	*p*-value
**TSS200-Island-cg02067788**	**1.607**	**0.011**
TSS200-Island-cg12202022	0.726	0.0069
TSS200-Island-cg23946014	1.374	0.072
**TSS200-Island-cg24159723**	**2.229**	**0.00017**
**TSS200-Island-cg26853048**	**1.902**	**0.0012**
**1stExon;5′URT-Island-cg04161464**	**1.601**	**0.011**
**1stExon;5′URT-Island-cg13778073**	**1.44**	**0.038**
Body-Island-cg16668042	1.332	0.17
TSS1500-Island-cg22771603	1.249	0.2
**Body-Open_Sea-cg05996250**	**1.836**	**0.0024**
**3′URT-Open_Sea-cg07474441**	**1.786**	**0.0035**
Body-S_shelf-cg10645426	0.757	0.17
TSS1500-N_Shore-cg11250576	0.724	0.073
TSS1500-N_Shore-cg12308504	1.678	0.13

**Note:**

Methylation CpG sites related to prognosis (*p* < 0.05) were presented in black font.

### Relationship between HSPA4 expression and immune cell infiltration

**Figure 8:**
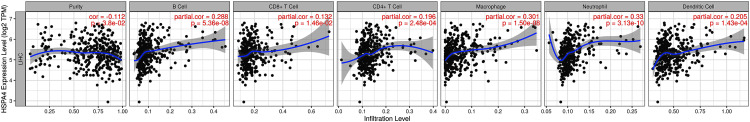
Relationship between HSPA4 expression and immune cell infiltration in HCC.

The relationship between HSPA4 expression and immune cell infiltration adjusted by purity (B cells, CD4+ T cells, CD8+ T cells, DCs, neutrophils, and macrophages) was investigated using TIMER. The results demonstrated that the expression level of HSPA4 positively correlated with infiltrating levels of B cells (r = 0.288, *p* = 5.36e−08), CD8+ T cells (r = 0.132, *p* = 1.46e−02), CD4+ T cells (r = 0.196, *p* = 2.48e−04), macrophages (r = 0.301, *p* = 1.50e−08), neutrophils (r = 0.33, *p* = 3.13e−10), and DCs (r = 0.205, *p* = 1.43e−04) in HCC but negatively correlated with tumor purity (r = −0.112, *p* = 3.8e−02) (Fig. 8).

### Correlation between HSPA4 expression and biomarkers of immune cells in HCC

To further explore the role of HSPA4 in the tumor immune micro-environment, we identified the correlation between HSPA4 expression and biomarkers of immune cells in HCC using the TIMER database (Table 2). As listed in Table 2, HSPA4 significantly positively correlated with B cell biomarkers (CD19, CD20, and CD38), CD8+ T cell biomarkers (CD8A), other T cell subsets (Tfh, Th1, Th2, Th9, Th17, Th22, and Treg), M1 macrophage biomarkers (IRF5 and PTGS2), M2 macrophage biomarkers (CD115), TAM biomarkers (PDCD1LG2, CD80, CD40, and TLR7), natural killer cell biomarkers (CD7 and XCL1), neutrophil biomarkers (ITGAM and FUT4), and dendritic cell biomarkers (CD1C and ITGAX) in HCC. These findings support that HSPA4 is positively linked to immune cell infiltration.

**Table 2:** Correlation analysis between HSPA4 expression and immune cell markers in HCC.

Immune cell	Biomarker	Cor	*p* value
**B cell**	CD19	0.132	*
	CD20 (KRT20)	0.227	***
	CD38	0.129	*
**CD8+ T cell**	CD8A	0.127	*
	CD8B	0.082	0.114
**Tfh**	BCL6	0.189	***
	ICOS	0.23	***
	CXCR5	0.147	**
**Th1**	T-bet (TBX21)	0.062	0.237
	STAT1	0.378	***
	STAT4	0.165	**
	IL12RB2	0.137	**
	WSX1 (IL27RA)	0.37	***
	IFN-γ (IFNG)	0.16	**
	TNF-α (TNF)	0.225	***
**Th2**	CCR3	0.371	***
	GATA3	0.149	**
	STAT5A	0.238	***
	STAT6	0.236	***
**Th9**	IRF4	0.107	*
	PU.1 (SPI1)	0.296	***
	TGFBR2	0.232	***
**Th17**	IL-17A	0.071	0.175
	IL-21R	0.228	***
	IL-23R	0.231	***
	STAT3	0.215	***
**Th22**	AHR	0.301	***
	CCR10	0.164	**
**Treg**	CCR8	0.348	***
	CD25 (IL2RA)	0.232	***
	FOXP3	0.102	0.05
**M1 macrophage**	COX2 (PTGS2)	0.197	***
	INOS (NOS2)	0.019	0.717
	IRF5	0.319	***
**M2 macrophage**	ARG1	−0.075	0.15
	CD206 (MRC1)	0.044	0.397
	CD115 (CSF1R)	0.252	***
**TAM**	PDCD1LG2	0.151	**
	CD80	0.284	***
	CD40	0.132	*
	TLR7	0.334	***
**Natural** **killer cell**	CD7	0.174	***
	KIR3DL1	−0.056	0.286
	XCL1	0.179	***
**Neutrophil**	CD11b (ITGAM)	0.35	***
	CD15 (FUT4)	0.291	***
	CD66b (CEACAM8)	0.092	0.076
**Dendritic cell**	CD1C	0.156	**
	CD11c (ITGAX)	0.334	***
	CD141 (THBD)	0.095	0.067

**Note:**

Tfh Follicular helper T cell, Th T helper cell, Treg Regulatory T cell, TAM Tumor-associated macrophage. *p < 0.05; **p < 0.01; ***p < 0.001.

### Relationship between HSPA4 expression and immune checkpoints in HCC

**Figure 9:**
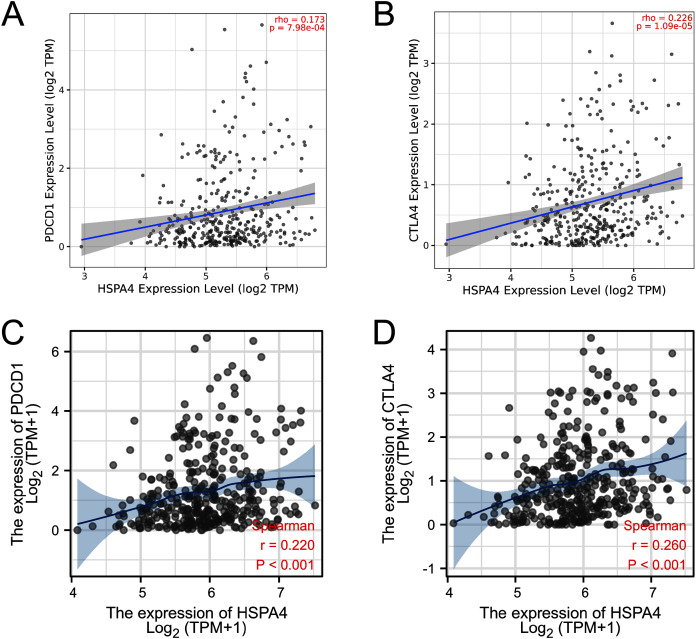
Correlation of HSPA4 expression with PD-1 and CTLA-4 expression in HCC. The correlation of HSPA4 expression with PD-1 (A, C) and CTLA-4 (B, D) in HCC by TIMER and TCGA databases, respectively.

PD-1 (PDCD-1) and CTLA-4 are important immune checkpoints that are responsible for tumor immune escape. Considering the potential oncogenic role of HSPA4 in HCC, the relationship of HSPA4 with PD-1 and CTLA-4 was assessed in both the TIMER and TCGA databases. There was a significant positive correlation of HSPA4 with PD-1 and CTLA-4 in HCC (Figs. 9A–9D).

### GO and KEGG pathway analyses

**Figure 10:**
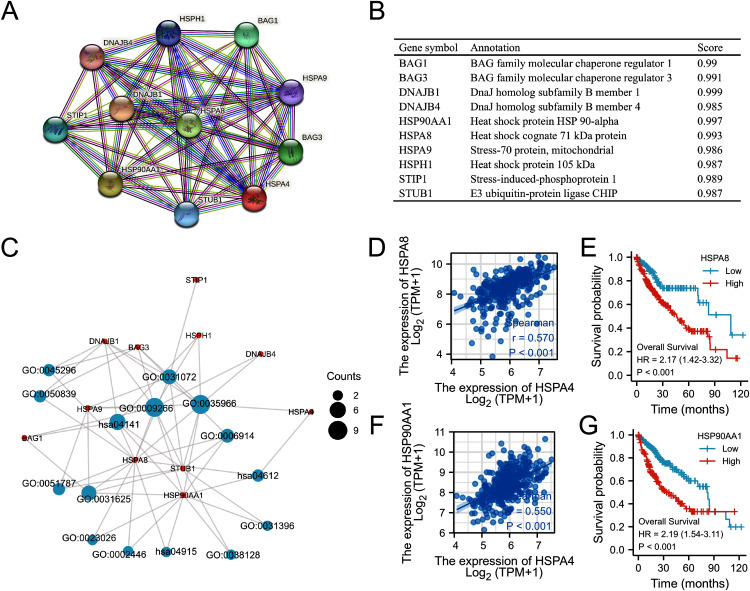
Functional annotations and predicted signaling pathways. (A) HSPA4-interaction proteins in HCC, visualized in a bubble chart. (B) Annotation of 10 functional partner genes of HSPA4. (C) GO term and KEGG pathway enrichment analyses. (D) The relevance between HSPA4 and HSPA8. (E) Survival analysis of HSPA8 in HCC. (F) The relevance between HSPA4 and HSP90AA1. (G) Survival analysis of HSP90AA1 in HCC.

Based on the STRING database, a PPI network of HSPA4 was constructed. The top ten functional partner genes were selected with a high degree of connectivity (Fig. 10A). These genes were *BAG1*, *BAG3*, *DNAJB1*, *DNAGB4*, *HSP90AA1*, *HSPA8*, *HSPA9*, *HSPH1*, *STIP1*, and *STUB1* (Fig. 10B). The KEGG pathway analysis revealed that *HSPA4*, *HSPA8*, and *HSP90AA1* were enriched in antigen processing and presentation (Fig. 10C; Table 3). The *HSPA4* expression level positively correlated with the levels of *HSPA8* (Fig. 10D) and *HSP90AA1* (Fig. 10F) in patients with HCC (r = 0.570, *p* < 0.001; r = 0.550, *p* < 0.001). Overexpression of *HSPA8* and *HSP90AA1* was also associated with a poorer prognosis in HCC (*p* < 0.001) (Figs. 10E, 10G). The GO enrichment analysis included three main functions, namely, biological process, cellular components, and molecular functions (*p* < 0.05) (Table 3). The biological process mainly included “response to temperature stimulus”, “response to topologically incorrect protein”, “autophagy”, and “regulation of protein ubiquitination”. Molecular functions mainly included “heat shock protein binding”, “ubiquitin protein ligase binding”, “misfolded protein binding”, “cadherin binding”, “cell adhesion molecule binding”, and “MHC class II protein complex binding”. Cellular components mainly included “chaperone complex”, “endocytic vesicle lumen”, “cytoplasmic vesicle lumen”, “inclusion body”, “lysosomal lumen”, and “fificolin-1-rich granule”.

**Table 3:** GO and KEGG enrichment analyses of HSPA4 and functional partner genes in HCC.

ONTOLOGY	ID	Description	*p* value
BP	GO:0035966	Response to topologically incorrect protein	8E−17
BP	GO:0009266	Response to temperature stimulus	4.97E−16
BP	GO:0006457	Protein folding	0.033424
BP	GO:0006914	Autophagy	0.00014
BP	GO:0038128	ERBB2 signaling pathway	0.000155
BP	GO:0031396	Regulation of protein ubiquitination	0.006124
CC	GO:0101031	Chaperone complex	3.81E−10
CC	GO:0071682	Endocytic vesicle lumen	5.35E−05
CC	GO:0060205	Cytoplasmic vesicle lumen	0.000744
CC	GO:0016234	Inclusion body	0.000917
CC	GO:0043202	Lysosomal lumen	0.001228
CC	GO:0101002	Ficolin-1-rich granule	0.004555
MF	GO:0031072	Heat shock protein binding	5.65E−09
MF	GO:0031625	Ubiquitin protein ligase binding	4.87E−07
MF	GO:0051787	Misfolded protein binding	4.61E-07
MF	GO:0023026	MHC class II protein complex binding	4.2E−05
MF	GO:0045296	Cadherin binding	0.000957
MF	GO:0050839	Cell adhesion molecule binding	0.003105
KEGG	hsa04141	Protein processing in endoplasmic reticulum	6.58E−09
KEGG	hsa04612	Antigen processing and presentation	6.98E−05

## Discussion

HSPA4 is expressed in various organs and can be induced by oncogenic stress (Kaneko et al., 1997). It is one of the HSP70 family members that facilitate cancer cell survival by inhibiting apoptosis and promoting proliferation (Saleh et al., 2000). Wu et al. (2011) reported that the knockdown of HSPA4 reduced the invasion and migration of tumor cells. In this study, we found that HSPA4 was significantly unregulated in most of the human cancers. Based on the TCGA and GEO databases and clinical samples, we verified overexpression of HSPA4 in HCC tissues compared with that in normal liver tissues. The expression of HSPA4 remarkably correlated with T stage, pathologic stage, histologic grade, and AFP level. Higher HSPA4 expression showed a worse prognosis and a more advanced cancer stage. HSPA4 expression showed a good ability to distinguish tumor from normal tissue, and to predict 1-, 3-, and 5-year survival rates, indicating that it can be used as a potential valuable diagnostic and prognostic biomarker for HCC.

Although gene mutations are closely related to tumors and often associated with a poor prognosis, the percentage of HSPA4 genetic alteration in HCC was only about 1.3%, and the genetic alteration showed no significant association with a poor OS. DNA methylation is a common epigenetic mechanism present in all forms of cancer. We investigated the relationship between DNA methylation levels of HSPA4 and the prognosis of HCC patients. Hypermethylation of seven CpG sites correlated with a worse OS, including cg05996250 and cg07474441, which presented the highest DNA methylation.

Treatment options for patients with HCC are limited, especially for those with advanced cancer. HCC is an inflammation-associated cancer, and the occurrence, progression, metastasis, and recurrence of HCC are closely related to immune response (Makarova-Rusher et al., 2015). Some immunotherapy approaches, including DC-based treatments, vaccines, immune checkpoint inhibitors, and adoptive cellular therapy, have been proven effective for some HCCs (Sangro et al., 2013; Li, Yang & Ren, 2015; El-Khoueiry et al., 2017). The importance of immunotherapy for HCC has been demonstrated by clinical trials and animal experiments (Zongyi & Xiaowu, 2020). Our study suggested that HSPA4 positively correlated with various immune cells, including B cells, CD8+ T cells, CD4+ T cells, macrophages, neutrophils, and dendritic cells in HCC. Moreover, HSPA4 also positively correlated with biomarkers of these infiltrated immune cells. In the HCC micro-environment, tumor-associated macrophages (TAMs) play an essential role in tumor growth, invasion, angiogenesis, and metastasis (Mantovani et al., 2011; Quail & Joyce, 2013; Noy & Pollard, 2014). We found that HSPA4 significantly correlated with TAMs. Regulatory T cells (Tregs) are associated with the invasiveness of HCC and are a promising independent predictor of recurrence and survival in HCC patients (Gao et al., 2007). We also found that HSPA4 positively correlated with Tregs. These results imply that HSPA4 may reflect the status of the immune micro-environment of HCC, and it plays an important role in immune regulation. Recently, immune checkpoint inhibitors (ICIs), including ipilimumab (the CTLA-4 inhibitor) and nivolumab (the PD-1 inhibitor), have demonstrated great survival benefits in patients with HCC (Waidmann, 2018; Lee et al., 2019). Furthermore, the efficacy of immunotherapy not only needs adequate immune cell infiltration into the tumor micro-environment but also depends on the sufficient expression of immune checkpoints (Chae et al., 2018). Thus, we also assessed the relationship between HSPA4 and immune checkpoints. The results demonstrated that the expression of HSPA4 positively correlated with PD-1 and CTLA-4 in HCC, indicating that targeting HSPA4 might increase the efficacy of immune checkpoint inhibitors in HCC.

Studies have indicated that because of inadequate antigen presentation by professional APCs such as DCs, the host immune system largely ignores or tolerates tumor antigens (Banchereau & Steinman, 1998). Recently, with the development of immunotherapy, DCs that present tumor-specific antigens to T cells have been developed as a vaccine to induce innate and adaptive immune responses. Here, KEGG pathway enrichment analysis revealed HSPA4 enrichment in antigen processing and presentation together with its functional partner HSPA8 and HSP90AA1 genes. Overexpression of HSPA8 and HSP90AA1 was also associated with a poorer prognosis in HCC. Previous studies have reported that HSPA8 and HSP90AA1 are associated with antigen processing and presentation (Song et al., 2010; Li et al., 2012; Chen et al., 2013; Deffit & Blum, 2015; Khosla et al., 2019). These results suggest that HSPA4, HSPA8, and HSP90AA1 have a synergistic effect in immunoregulation and tumor promotion.

GO enrichment analysis showed that HSPA4 and interacted genes not only participate in the basic biological process of response to temperature stimulus but also participate in tumorigenesis and progression, such as autophagy, regulation of protein ubiquitination, cadherin binding, cell adhesion molecule binding, and MHC class II protein complex binding. Autophagy has been implicated as a process that regulates cancer (Amaravadi, Kimmelman & Debnath, 2019). Ubiquitination plays a crucial role in ensuring cell homeostasis and guaranteeing life activities, and the aberrant regulation of ubiquitination could induce various types of cancers (Deng et al., 2020). The downregulation of cadherin and cell adhesion molecule binding correlated with invasive states, and it is essential to cancer progression (Ito et al., 2017). MHC-II is expressed by antigen-presenting cells or tumor cells (Kambayashi & Laufer, 2014), presenting exogenously derived peptide antigens to CD4+ T cells. Tumor-specific MHC-II expression may increase recognition of a tumor by the immune system (Axelrod et al., 2019). These findings confirmed the role of HSPA4 in HCC occurrence and progression and verified the correlation between HSPA4 and immune regulation of HCC.

There were some limitations to this study. First, the immune status underlying immunogenetic changes requires further exploration, as it was only estimated in our study. Second, the functional analysis of HSPA4 in immunoregulation requires further research.

## Conclusions

In this study, we verified the value of HSPA4 in the diagnosis and predicting prognosis of HCC. DNA methylation of HSPA4 is related to the prognosis of HCC. HSPA4 may not only participate in the occurrence and progression but also the immune regulation of HCC. Therefore, HSPA4 can be a potential diagnostic and prognostic biomarker and a therapeutic target for HCC. The prediction of HSPA4 functions provided further insights into the pathogenesis of HCC and the role of HSPA4 in immunotherapy for HCC. Further mechanistic studies are needed to validate our findings and promote clinical application.

## Supplemental Information

10.7717/peerj.12315/supp-1 Supplemental Information 1 Clinical data of the LIHC patients from TCGA.

10.7717/peerj.12315/supp-2 Supplemental Information 2 Clinical information of HCC patients participating in immunohistochemical staining.

## Additional Information and Declarations

### Data Availability

The following information was supplied regarding data availability: The raw measurements are available in the Supplementary Files.
