# Supplementary material for: Significant correlation between HSPA4 and prognosis and immune regulation in hepatocellular carcinoma
Source: PeerJ. 2021 Oct 26;9:e12315. doi: 10.7717/peerj.12315 (PMC8555498; doi:10.7717/peerj.12315)
Supplement: Supplemental Information 2 [file peerj-09-12315-s002.docx]

| Case | Sex | Age | Diagnosis | Date of diagnosis |
| --- | --- | --- | --- | --- |
| Case 1 | Man | 73 | HCC | 2021.5.16 |
| Case 2 | Man | 63 | HCC | 2021.5.31 |
| Case 3 | Man | 77 | HCC | 2021.5.14 |
| Case 4 | Man | 49 | HCC | 2021.5.17 |
| Case 5 | Man | 61 | HCC | 2021.5.24 |

Clinical information of HCC patients participating in immunohistochemical staining staining
